# Education of the Judgment

**Published:** 1897-05-22

**Authors:** 


### Education of the Judgment.

The parable of the sower remains as true, and the waste of good seed by the wayside, or in stony places, or among thorns, is as flagrant to-day as when the lesson to be learnt from these occurrences was first impressed upon mankind. The latest example has perhaps been furnished by Lord Rosebery, who has recently gained much praise if measured by quantity, although somewhat less if estimated by value, by his possibly obvious announcement that current opinion, if its effects are to be otherwise than harmful, require to be carefully revised by the exercise of mature judgment, and by his remarkable dictum that the power of forming such a judgment is to be obtained from the study of enduring as well as of ephemeral literature, and also by intercourse and discussion with our fellow men. The good seed to which, in this particular instance, we refer, and which seems to have shared the usual fate of such material, was sown in the year 1854 by no less a person than Michael Faraday, in a lecture on mental education which he delivered at the Royal Institution of Great Britain. The burden of his complaint was not only the prevailing "deficiency of judgment," but also the want of any endeavour, as a matter of ordinary training, to educate the faculty to the level of its responsibilities. "I know," he said, "that in physical matters multitudes are ready to draw conclusions who have little or no power of judgment in the cases; that the same is true of other departments of knowledge; and that, generally, mankind is willing to leave the faculties which relate to judgment almost entirely uneducated, and their decisions at the mercy of ignorance, prepossessions, the passions, or even accident." In continuation of his subject, he pointed out, with the eloquence and force characteristic of his greatest efforts, the ill consequences which flow from non-recognition of the almost universal deficiency of judgment among persons who, if measured by any standard of literary attainment, would be described as well educated, but who, mainly because they have never been taught to realize their incapacity, have never by any serious endeavour attempted to overcome it.

Faraday held it to be as much a duty, on the part of every man, to aim at the improvement of his own judgment, as it is the duty of a parent to send his child to school. He did not suggest as an agency for the purpose either books or gossip; but the habitual practice of criticising one's own conclusions, in such a way as to ascertain how far they have been verified by events, and on what points and to what extent they have been erroneous. He made no claim to a monopoly of sound judgment as a privilege of philosophers; but expressed his conviction that, although a powerful mind will be able to draw a conclusion more readily and more correctly than one of moderate character, it is more or less in the power of every man greatly to improve himself in this respect.

We boast, and, perhaps, not without reason, of the nineteenth century and of the reign of Victoria; but it is impossible not to see that education of the judgment, if, even in the most moderate degree, it could be made universal, or even general, would be likely to bring in its train advantages which it would be impossible for us, with our present surroundings, even to estimate by conjecture. Let us only remember that, if brought before the tribunal of a trained judgment, all quackery, whether medical, religious, or political, would be tried in the balance and rejected as unworthy of trust; with the primary result that our statesmen and Parliaments would be delivered from bondage to fools, and would be able to apply themselves with single minds to the initiation of such changes, or to the preservation of such institutions, as would certainly be conducive to the welfare of the community. Even the extinction of the prevailing belief in quack medicines, and the consequent abandonment of the existing practice of paying a shilling for a farthing's worth of jalap, because it is advertised as Jones's pill, would, it is said, save three millions a year to the community, or be equivalent to a reduction of twopence in the income tax. At present, unfortunately, the tendency of what is called education, at all events of the education of the majority, is distinctly unfavourable to the realisation of that Utopia which the very phrase, exercise of the judgment, calls up before even the most disciplined imagination. The ordinary effect of schooling is to convince children how clever they are; and distrust of self is the last thing likely to be inculcated by an average teacher. Faraday was before his time; and he spoke to a public who found it difficult to believe that intellectual modesty is the beginning of wisdom. But perhaps we have made some progress, and it may even be that Lord Rosebery, with his prescription of books and gossip, is lagging somewhat in the rear of the higher planes of contemporary intelligence. However this may be, he at any rate appears to be fully abreast of no small number of our contemporaries.

